# ﻿Species delimitation and DNA barcoding for Chinese Mantodea (Insecta, Dictyoptera)

**DOI:** 10.3897/zookeys.1229.129123

**Published:** 2025-02-24

**Authors:** Guangju Yang, Wenjing Wang, Yuwei Tong, Zhijun Zhou

**Affiliations:** 1 Key Laboratory of Zoological Systematics and Application of Hebei Province, College of Life Sciences, Hebei University, Baoding, Hebei 071002, China Hebei University Baoding China; 2 Hebei Basic Science Center for Biotic Interaction, Institute of Life Science and Green Development, Hebei University, Baoding, Hebei 071002, China Hebei University Baoding China

**Keywords:** China, COI-5P, DNA barcoding, mantis, Mantodea, species delimitation

## Abstract

DNA barcoding has been proposed as a rapid and reliable tool for animal identification and species delineation. The 5’ end of the mitochondrial cytochrome *c* oxidase I gene (COI-5P) was sequenced for 318 specimens of 55 mantis species. Of these, 44 species had not been sequenced before, thus being new COI-5P barcode sequences to science. Another 61 COI-5P barcode sequences comprising five species were retrieved from the Barcode of Life Database (BOLD; www.boldsystems.org). Five species delimitation algorithms were employed to sort barcode sequences into Molecular Operational Taxonomic Units (MOTUs), namely the distance-based Barcode Index Number (BIN) System, Generalized Mixed Yule Coalescent (GMYC), a Java program that uses an explicit, determinate algorithm to define Molecular Operational Taxonomic Unit (jMOTU), Assemble Species by Automatic Partitioning (ASAP), and Bayesian implementation of the Poisson Tree Processes model (bPTP). All species, except *Hierodulachinensis* Werner, 1929, were recovered as monophyletic on the neighbor-joining (NJ) tree. For the final dataset, 379 COI-5P barcode sequences were assigned to 68 BINs. Fifty-five out of 68 BINs obtained were new to BOLD. The low level of BIN overlap with other nations highlights the importance of constructing a regional DNA barcode reference library. The algorithms ASAP, jMOTU, bPTP, and GMYC clustered barcode sequences into 32, 58, 68, and 60 MOTUs, respectively. All species delimitation algorithms (except ASAP analysis) split *Anaxarchasinensis* Beier, 1933, *Anaxarchazhengi* Ren & Wang, 1994, *H.chinensis*, *Spilomantisoccipitalis* (Westwood, 1889), *Titanodulaformosana* Giglio-Tos, 1912 into more than one MOTUs. All algorithms merged *Hierodula* sp. BCM-2019 and *H.chinensis* into the same MOTU, as for *Tenoderaaridifolia* Stoll, 1813 and *Tenoderasinensis* Saussure, 1871. More accurate identification results need to be supplemented by detailed morphological classification.

## ﻿Introduction

Correct taxonomic identification and species delimitation is critical to the field of biology (de Queiroz 2007). Morphological taxonomic identification is time-consuming, requires (increasingly rare) taxonomic expertise, and is dependent on the integrity of the external characteristics of the specimens. Taxonomic keys are usually only valid for a particular life stage or gender, so many individuals are unrecognizable (Hebert et al. 2003a). Many insect species await descriptions and some named taxa represent a species complex (Bickford et al. 2007). Taxonomic ambiguities and uncertainties frequently occur because of cryptic or hidden undescribed species (Lobo et al. 2016). These limitations have led to the need for a new approach to species recognition (Fišer Pečnikar and Buzan 2014; Porter et al. 2014).

A 650 bp fragment of the 5’ end of the mitochondrial cytochrome *c* oxidase I gene (COI-5P) was proposed as a standardized barcode region for animals (Hebert et al. 2003a). During the last 20 years, COI-5P barcode sequences have been used as a rapid tool for specimen identification, species delineation, and discrimination of closely related species (Hebert et al. 2004; Dupuis et al. 2012; Zhou et al. 2019). Many studies have reported the utility of using DNA barcoding in different insect orders, such as Coleoptera (Oba et al. 2015), Diptera (Chan et al. 2014; Pinto et al. 2023), Hemiptera (Khamis et al. 2017), Lepidoptera (Hebert et al. 2004; Huemer et al. 2020), and Orthoptera (Hawlitschek et al. 2017; Zhou et al. 2019). Critical for DNA barcoding identification is the degree of taxonomic coverage of these libraries. So far, relatively comprehensive DNA barcode libraries for several well-known insect groups have been reported (Khamis et al. 2017; Moriniere et al. 2017; Huemer et al. 2020; Posada-Lopez et al. 2023). Virgilio et al. (2012) have modelled relationships between identification performance and distance thresholds and proposed a general working strategy for identifying species when DNA barcode libraries are incomplete.

The Barcode of Life Data (BOLD) system (https://www.boldsystems.org) is an informatics workbench aiding the acquisition, storage, analysis, and publication of DNA barcoding records (Ratnasingham and Hebert 2007). Several species delimitation algorithms have been designed to sort DNA barcode sequences into Molecular Operational Taxonomic Units (MOTUs) without a priori information. They can be classified into two categories: (1) similarity-based methods, for example, Barcode Index Number (BIN) (Ratnasingham and Hebert 2013), Automatic Barcode Gap Discovery (ASAP), a hierarchical clustering algorithm that only uses pairwise genetic distances (Puillandre et al. 2021) and a Java program that uses an explicit, determinate algorithm to define Molecular Operational Taxonomic Unit (jMOTU) (Jones et al. 2011); and (2) tree-based methods, for example, Bayesian implementation of the Poisson Tree Processes model (bPTP) (Zhang et al. 2013) and Generalized Mixed Yule Coalescent (GMYC) (Pons et al. 2006; Fontaneto et al. 2007). These algorithms greatly help in the revision of species incorrectly described by conventional morphology (Renaud et al. 2012).

Mantodea is a predatory insect group that occupies an important ecological niche and occurs in a variety of habitats, such as tropical rainforests, arid forests, and deserts (Zhang and Ye 2017; Mirzaee et al. 2024). The Mantodea Species File (MSF) records 2514 extant valid species and 164 subspecies worldwide distributed in 29 families, with many species yet to be described (Otte et al. 2024). Until now, only a few molecular identification studies on Mantidis Oötheca (mantis egg case; Sangpiaoxiao) using DNA barcoding have been reported (Wang et al. 2015; Song et al. 2020). The BOLD system records show 2990 published mantis barcode records from 63 countries forming 723 BINs, of which 1289 records have species names and represent 417 species (last accessed on 15 April 2024). The Chinese Mantodea fauna is remarkably rich with many endemic species. Progress in DNA barcoding the described fauna of Chinese Mantodea lags far behind most countries. As of 15 April 2024 (excluding the current study) there were 61 barcode records from China forming 13 BINs, all mined from GenBank, NCBI, according to a search of the BIN database in the public portal of BOLD. This study presents the first results of DNA barcoding of mantis from China. The main aims of the study are to compare the generated data with existing data (assign unknown specimens to species); and enrich the existing DNA barcode reference library with new sequences of specimens collected in China.

## ﻿Materials and methods

### ﻿Taxon sampling

A total of 318 mantis specimens were collected from 13 provinces in China, and preserved in absolute alcohol. Every individual was identified morphologically by the authors to the finest taxonomic level possible. The identification results include 43 named species and another 12 species that presently can only be reliably identified to genus level (Table 1). All specimens were photographed, labeled, and individually stored at − 20 °C prior to DNA extraction.

**Table 1.** The distribution of K2P sequence divergence at each taxonomic level.

Taxonomic level	Taxa	Comparisons	Min. Dist. (%)	Max. Dist. (%)	Mean Dist. (%)
Within Species	34	3495	0.00	7.63	0.98
Within Genus	16	5485	0.31	17.06	8.62
Within Family	4	17191	3.46	22.69	14.67

### ﻿DNA extraction, PCR amplification, and sequencing

DNA was extracted from muscle tissue of the leg using the TIANamp Genomic DNA kit (Tiangen Biotech, Beijing, China), following the manufacturer’s instructions. Polymerase chain reaction (PCR) amplification followed the method of Zhou et al. (2019), with COBU (5’-TYT CAA CAA AYC AYA ARG ATA TTG G-3’) and COBL (5’‐TAA ACT TCW GGR TGW CCA AAR AAT CA‐3’) as primers (Pan et al. 2006). Sanger sequencing of PCR products was carried out in both directions at Genewiz (Tianjin, China). DNA sequences were checked, edited, and assembled into consensus sequences using Seq Man (Burland 2000). Consensus sequences were translated to amino acid sequences to check for stop codons (but none were found) in Editseq. Obtained sequences, along with the collection data, images, and trace files, are deposited in the BOLD systems.

### ﻿Data analyses

To obtain DNA barcodes, we searched for public barcode sequences for Mantodea of China in the Barcode of Life Database (BOLD; www.boldsystems.org). In total, 61 public barcode sequences were retrieved for analysis, which represented three named species (e.g., *Hierodulapatellifera* Serville, 1839, *Leptomantellaalbella* (Burmeister, 1838), *Tenoderasinensis* Saussure, 1871) and another two species (e.g., *Hierodula* sp. BCM-2019, *Tenodera* genus) which presently can only be reliably identified to genus level. All newly generated barcode sequences were given a Barcode Index Number (BIN) on the BOLD platform (Ratnasingham and Hebert 2013). Sequence divergences were ascertained using the “Distance Summary” and “Barcode Gap Analysis” tools on the BOLD platform (Ratnasingham and Hebert 2007). All sequences meeting the required quality criteria (sequence record > 500 bp with < 1% Ns, no stop codon, no contamination or error flag, and with trace files available, and voucher specimen with at least country of origin) were aligned using the BOLD Aligner (Amino Acid based HMM) algorithm (Eddy 1998; Ratnasingham and Hebert 2013; Zhang et al. 2024). The Kimura 2-parameter (K2P) (Kimura 1980) was used as the phylogeny model and uncorrected pairwise distance (p-distance) was employed for all distance calculations (Ratnasingham and Hebert 2013). The Taxon ID Tree was constructed under the K2P distance model using the neighbor-joining algorithm in BOLD and visualized using Figtree v. 1.4.4 (available at http://tree.bio.ed.ac.uk/software/Figtree/).

We also employed four algorithms (ASAP, jMOTU, bPTP, and GMYC) to sort COI-5P barcode sequences into MOTUs. All the new and public COI-5P barcode sequences were downloaded and collapsed into 204 unique haplotypes using DnaSP v. 6.0 (Rozas et al. 2017). ASAP analysis was run on the web server at https://bioinfo.mnhn.fr/abi/public/asap using three distance models (Jukes-Cantor, K2P, and Simple distance) with default parameters (Puillandre et al. 2021). jMOTU analysis was run using threshold values initially from 1 to 40 bp, covering a range of 0.15% to 6.08% divergence with respect to the full-length (658 bp) COI-5P barcode sequences (Jones al et. 2011). For bPTP, the input Bayesian inference (BI) tree was obtained in Phylosuite v.,1.1.2 (Zhang et al. 2020). Tracer v.,1.7.1 (Rambaut et al. 2018) was used to check the convergence of these trees (effective sample size (ESS) > 200). We ran this BI tree for 500,000 MCMC generations using the online server (http://species.h-its.org/) with a burn-in of 0.1, set as a rooted tree, excluding outgroups, and with other parameters set as default. For GMYC, ultrametric trees were reconstructed using BEAST v.,1.7 (Drummond and Rambaut 2007), with the GTR+F+G4 substitution model. Splits (Ezard et al. 2009) and Ape (Paradis et al. 2004) were used for single-threshold GMYC (sGMYC) (Pons et al. 2006) analysis.

## ﻿Results

### ﻿Overview

For this study, we obtained 318 COI-5P barcode sequences from 43 named species and another 12 species that could only be reliably identified to genus level. Of these, 44 species had not been sequenced before, thus being new COI-5P barcode sequences to science. In addition, we also sequenced previously published species for the COI-5P gene, but which are new to China: *Anaxarchazhengi* Ren & Wang, 1994, *Creobrotergemmatus* (Stoll, 1813), *Eomantisyunnanensis* Wang, 1993, *Hierodulachinensis* Werner, 1929, *Mantisreligiosa* (Linne, 1758), *Statiliamaculata* Thunberg, 1784, *Statilianemoralis* Saussure, 1870, *Tenoderaangustipennis* Saussure, 1869, and *Titanodulaformosana* (Giglio-Tos, 1912). All sequences complied with the barcode standard described in BOLD (http://www.boldsystems.org). None of the sequences was flagged, which indicated that there were no problematic records. In addition, 61 published barcode sequences representing five species were also retrieved for analysis. All data are available in BOLD through the public dataset DS-DBMC (DNA Barcode Library for Mantodea of China). The final dataset consisted of 379 COI-5P barcode sequences (204 unique haplotypes) from 57 mantis species, of which 14 were assigned only at the genus level. The number of barcode sequences per species ranged from one (22 species) to a maximum of 52 for *H.chinensis*. Most subsequent species delimitation analyses were performed on the haplotype dataset.

**Figure 1.**
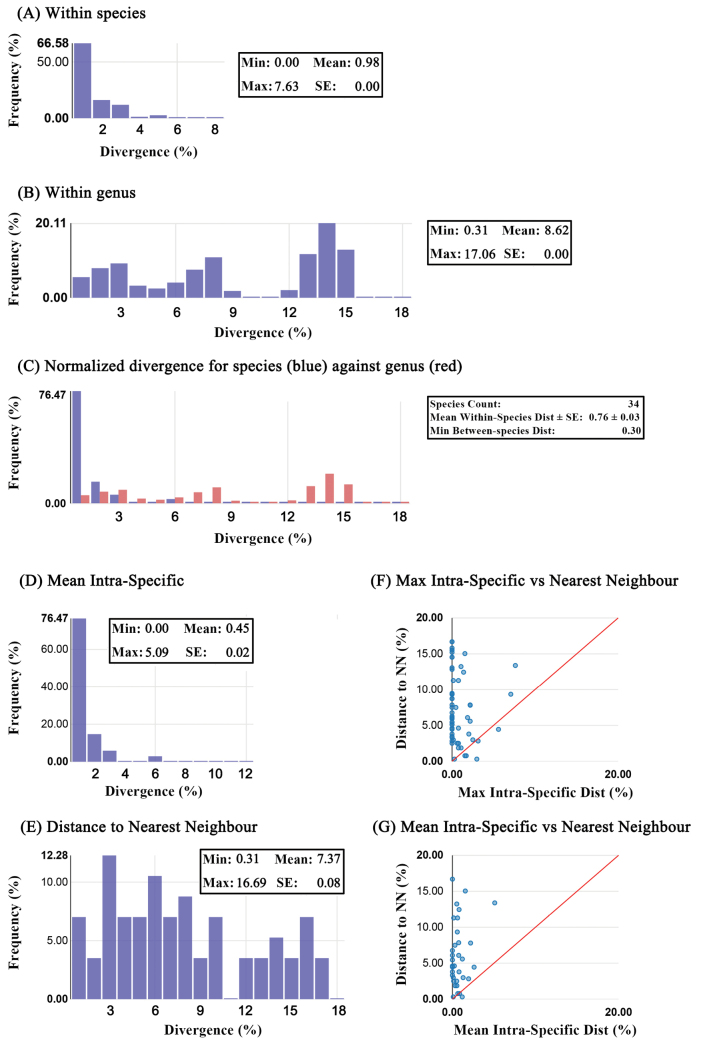
Inter- and intraspecific genetic divergence based on K2P distances. **A** Distance distribution histograms of mean intra-specific distances **B** distance distribution histograms of distance to nearest neighbor **C** maximum intra-specific vs nearest-neighbor distances **D** mean intraspecific vs nearest-neighbor distances **E** number of individuals in each species against their maximum intraspecific distances.

The distribution of K2P sequence divergence for each taxonomic level is summarized in Table 1. The mean intraspecific K2P divergence was 0.98% (range 0−7.63%) while the mean congeneric divergence was 8.62% (range 0.31−17.06%), and mean divergence within a family was 14.67% (range 3.46−22.69%). The normalized mean intraspecific and minimum interspecific distance were 0.76 ± 0.03 and 0.31%, respectively. The mean and maximum intraspecific divergence ranged between 0−5.09% and 0−7.63%, respectively (Table 2). The nearest-neighbor (NN) distance ranged from 0.31% (*H.chinensis* vs. *Hierodula* sp. BCM-2019) to 16.69% (*Eomantisguttatipennis* Stal, 1877 vs *E.yunnanensis*). Deep intraspecific divergences (> 2%) overlapping with DNN were detected in 10 species, viz., *Acromantishesione* Stal, 1877 (2.5%), *Anaxarchasinensis* Beier, 1933 (5.59%), *A.zhengi* (7.08%), *C.gemmatus* (2.18%), *H.chinensis* (2.98%), *Statiliaflavobrunnea* Zhang, 1984 (2.02%), *Spilomantisoccipitalis* (Westwood, 1889) (7.63%), *T.formosana* (3.14%), *Theopompamaculosa* Yang, 1997 (2.18%), and *Theopropus* sp. 1 WJ-2021 (2.19%). (Table 2). Fig. 1A, B shows the distance distribution histograms of mean intraspecific distances and distance to nearest neighbor (NN), respectively. Fig. 1C, D shows overlap of the maximum and mean intra-specific distances and distance to the nearest neighbor (NN), respectively. Fig. 1E shows the distribution of normalized divergence for species (blue) against the genus-level divergence (red).

**Table 2.** Nominal species, mean and maximum intraspecific divergence, and the minimum distance to the nearest neighbor (NN) of mantis species from China. BIN, Barcode Index Number; *N*, number of barcodes per BIN; *I_mean_*, mean intraspecific distance; *I_max_*, maximum intraspecific distance; DNN, distance to nearest neighbor; species in bold and labelled* *I_max_*> DNN. * Species that had their COI-5P barcode sequenced for the first time;”Warning species” those where the distance to nearest neighbor (NN) is less than 2% divergent, or when the distance to NN is less than the maximum intraspecific distance are highlighted in bolded.

Species	BIN (*N*)	*I*_mean_	*I*_max_	Nearest neighbor (NN) species	Distance to NN
*Pseudempusapinnapavonis* Brunner, 1893*	^N^AEI4750 (1)	N/A	0	*Statiliaflavobrunnea*	15.81
*Amantiswuzhishana* Yang, 1997*	^N^AEM6545 (5)	0.52	1.08	*Gonypetabrunneri*	13.23
*Gonypetabrunneri* Giglio-Tos, 1915*	^N^AEN4222 (3)	0.62	0.77	*Gonypeta* sp. WJ-2021	11.29
*Gonypeta* sp. WJ-2021*	^N^AEM8003 (2)	0.15	0.15	*Gonypetabrunneri*	11.29
*Spilomantisoccipitalis* (Westwood, 1889)*	^N^AEI3640 (3)	5.09	7.63	*Amantiswuzhishana*	13.39
	^N^AEI3638 (1)				
	^N^AEI3641 (1)				
	^N^AEI3639 (1)				
*Theopompamaculosa* Yang,1997*	^N^AEN1268 (6)	0.75	2.18	*Theopompaophthalmica*	7.84
*Theopompaophthalmica* Olivier, 1792*	^N^AEN3171 (1)	N/A	0	*Theopompamaculosa*	7.84
*Arriabrevifrons* (Wang, 1991)*	^N^AEM8412 (1)	N/A	0	*Arriasticta*	8.72
*Arriapura* Wang & Chen, 2021*	^N^AEI6929 (1)	N/A	0	*Arriabrevifrons*	9.32
*Arria* sp. WJ-2021*	^N^AEI6928 (1)	N/A	0	*Arriabrevifrons*	13.07
*Arriasticta* (Zhou & Shen, 1992)*	^N^AEM8413 (1)	N/A	0	*Arriabrevifrons*	8.72
*Caliris* sp. WJ-2021*	^N^AEI1888 (1)	N/A	0	*Odontomantis* sp. WJ-2021	15.22
*Sinomiopteryx* sp.2 WJ-2021*	^N^AEI6930 (1)	N/A	0	*Sinomiopteryx* sp. 1 WJ-2021	15.52
*Sinomiopteryx* sp. 1 WJ-2021*	^N^AEI6931 (2)	1.55	1.55	*Anaxarcha* sp.	15.04
*Acromantishesione* Stal, 1877*	^N^AEM9742 (2)	1.29	2.5	*Acromantisjaponica*	2.98
	^N^AEM9743 (5)				
	^N^AEM9741 (1)				
*Acromantisjaponica* Westwood, 1889*	^N^AEM5713 (3)	0.1	0.15	*Acromantishesione*	2.98
*Anaxarchagraminea* Stal, 1877*	^N^AEN5722 (12)	0.15	0.62	*Anaxarchatianmushanensis*	2.5
*Anaxarchasinensis* Beier, 1933*	^N^AEI0968 (2)	2.61	5.59	*Anaxarchatianmushanensis* DBMC250-21	4.44
	^N^AEI0969 (3)				
	^N^AEI0967 (14)				
	^N^AEN2989 (4)				
*Anaxarcha* sp.*	^N^AEN1986 (2)	0	0	*Anaxarchagraminea*	3.31
*Anaxarchatianmushanensis* Zheng, 1985*	^N^AEN5442 (1)	N/A	0	*Anaxarchagraminea*	2.5
*Anaxarchazhengi* Ren & Wang, 1994	ADR8634 (27)	0.58	7.08	*Odontomantis* sp. WJ-2021	9.34
	^N^AEI4467 (1)				
*Astyliasulamajor* (Beier, 1929) *	^N^AEI8570 (1)	N/A	0	*Acromantishesione*	12.84
*Creobrotergemmatus* (Stoll, 1813)	ADR7829 (4)	1.2	2.18	*Creobrotervitripennis*	5.58
	^N^AEM7118 (4)				
*Creobroternebulosa* Zheng, 1988 *	^N^AEI7150 (8)	0.23	0.77	*Creobrotervitripennis*	4.61
*Creobrotervitripennis* Beier, 1933*	^N^AEI7149 (2)	0	0	*Creobroternebulosa*	4.61
*Odontomantisplaniceps* Haan, 1842*	^N^AEN4786 (1)	N/A	0	*Odontomantis* sp. WJ-2021	9.46
*Odontomantis* sp. WJ-2021*	^N^AEI1478 (1)	N/A	0	*Anaxarchazhengi*	9.34
*Phyllothelyssinensis* Ouchi, 1938*	^N^AEI2011 (3)	0.31	0.46	*Phyllothelyswerneri*	7.49
*Phyllothelyswerneri* Karny, 1915*	^N^AEN4577 (1)	N/A	0	*Phyllothelyssinensis*	7.49
*Theopropussinecus* (Yang, 1999) *	^N^AEH9000 (1)	N/A	0	*Theopropus* sp. 2 WJ-2021	5.12
*Theopropus* sp. 1 WJ-2021*	^N^AEH8998 (1)	2.19	2.19	*Theopropus* sp. 2 WJ-2021	7.8
	^N^AEH8999 (1)				
*Theopropus* sp. 2 WJ-2021*	^N^AEN8032 (1)	N/A	0	*Theopropussinecus*	5.12
*Leptomantellaalbella* (Burmeister, 1838)	ADC8427 (1)	N/A	0	*Leptomantellatonkinae*	5.44
*Leptomantella* sp. WJ-2021*	^N^AEI0541 (1)	N/A	0	*Leptomantellaxizangensis*	14.54
*Leptomantellatonkinae* Hebard, 1920*	^N^AEN5841 (4)	0	0	*Leptomantellaalbella*	5.44
*Leptomantellaxizangensis* Wang, 1993*	^N^AEM5629 (1)	N/A	0	*Leptomantella* sp. WJ-2021	14.54
*Hierodulachinensis* Werner, 1929	ADC1760 (30)	1.18	2.98	*Hierodula* sp. BCM-2019	0.31
	AEI8831 (22)				
*Hierodulalatipennis* Brunner, 1893*	^N^AEM5843 (1)	N/A	0	*Titanodula* sp. WJ-2021	6.26
*Hierodulalonga* (Yang, 1997)*	^N^AEI1830 (2)	0	0	*Hierodulachinensis*	4.46
*Hierodulamaculata* Wang, Zhou & Zhang, 2020*	^N^AEM5027 (2)	0	0	*Hierodulapatellifera*	6.08
*Hierodulapatellifera* Serville, 1839	ACD7790 (20)	0.76	1.86	*Hierodulamaculata*	6.08
*Hierodula* sp. BCM-2019	AEI8831 (12)	0.1	0.29	*Hierodulachinensis*	0.31
*Hierodulazhangi* Wang & Dong, 1993*	^N^AEI9445 (1)	N/A	0	*Titanodula* sp. WJ-2021	3.4
*Mantisreligiosa* (Linne, 1758)	AAF4833 (5)	0.8	1.39	*Tenoderasinensis*	12.45
*Rhomboderalatipronotum* Zhang, 1990*	^N^AEI5803 (1)	N/A	0	*Titanodula* sp. WJ-2021	5.91
*Statiliaagresta* Zheng, 1987*	^N^AEI8679 (4)	0.54	0.77	*Statiliamaculata*	1.86
*Statiliaflavobrunnea* Zhang, 1984*	ADR8864 (17)	0.79	2.02	*Statilianemoralis*	3.79
*Statiliamaculata* Thunberg, 1784	ACD7572 (27)	0.33	1.09	*Statiliaagresta*	1.86
*Statilianemoralis* Saussure, 1870	ACR3531 (2)	0	0	*Statiliaflavobrunnea*	3.79
*Tenoderaangustipennis* Saussure, 1869	ADK7863 (31)	0	0	*Tenoderaaridifoliabrevicollis*	6.75
*Tenoderaaridifolia* Stoll, 1813*	AAW5350 (16)	0.85	1.71	*Tenoderasinensis*	0.77
*Tenoderasinensis* Saussure, 1871	AAW5350 (30)	0.64	1.55	***Tenoderaaridifoliabrevicollis***	0.77
*Titanodula* sp. WJ-2021*	^N^AEM8605 (1)	N/A	0	*Titanodulaformosana*	2.82
***Titanodulaformosana* Giglio-Tos, 1912**	^N^AEN7193 (2)	1.94	3.14	***Titanodula* sp. WJ-2021**	2.82
	^N^AEN7194 (2)				
*Tenodera* sp. WJ-2021*	^N^AEI9742 (3)	0.51	0.77	*Tenoderaaridifoliabrevicollis*	2.5
*Eomantisguttatipennis* Stal, 1877*	^N^AEI9798 (1)	N/A	0	*Eomantisyunnanensis*	16.69
*Eomantisyunnanensis* Wang, 1993	ADW8794 (2)	0	0	*Eomantisguttatipennis*	16.69

### ﻿Barcode index numbers (BINs) and MOTUs estimation

**Figure 2.**
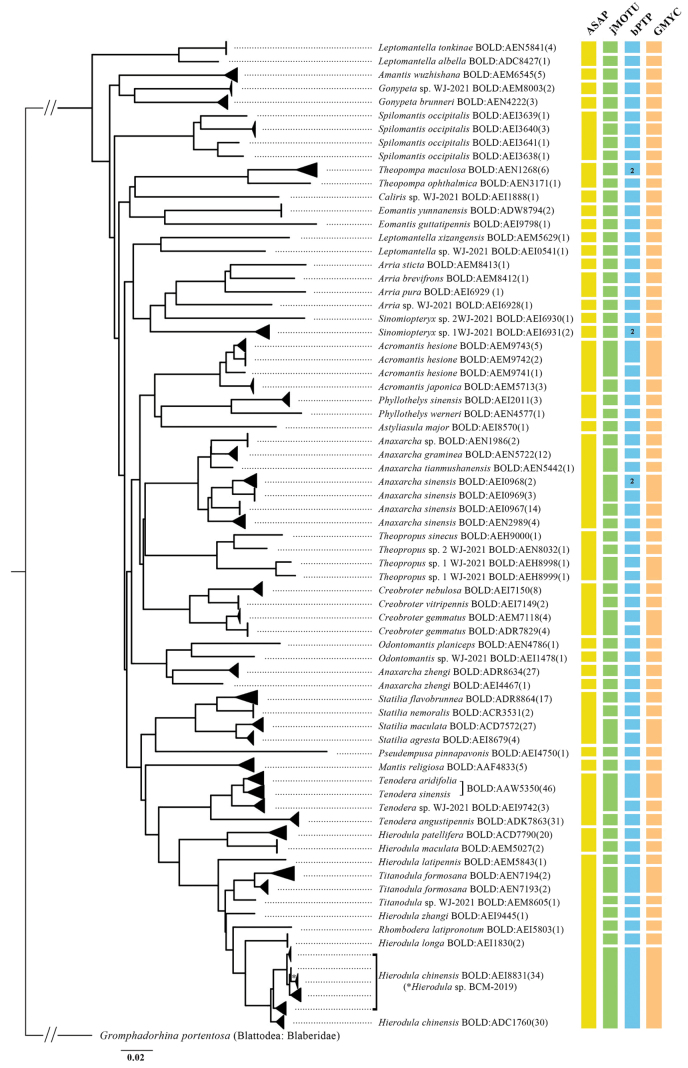
Taxon ID Tree of mantis COI-5P barcode sequences using the neighbor-joining (NJ) method based on K2P distances (left) and groups showing contrasting species delimitation results (right). The scale bar shows K2P distances. Numbers in the rectangles indicate the number of MOTUs.

The BIN analyses were performed on 15 April 2024. All sequences qualified for BIN analysis. In total, 379 sequences were assigned to 68 different BINs and 55 (80.0%) were new to BOLD. Fifty-four of 57 species had a unique BIN or were assigned to more than one BIN that formed single clades allowing unambiguous identification based on DNA barcodes. Thirty BINs were represented by a single record (Fig. 2, Table 2). Two species were lumped together by BIN algorithms in two cases, viz., *T.sinensis* vs. *T.aridifolia* (BOLD: AAW5350) and *H.chinensis* vs. *Hierodula* sp. BCM-2019 (BOLD: AEI8831). More than one BIN was associated with eight species, with up to eight BINs for some, including *A.hesione* (BOLD: AEM9742, AEM9743, AEM9741), *A.sinensis* (BOLD: AEI0967, AEI0968, AEI0969, AEN2989), *A.zhengi* (BOLD: ADR8634, AEI4467), *C.gemmatus* (BOLD: AEM7118, ADR7829), *H.chinensis* (BOLD: ADC1760, AEI8831), *S.occipitalis* (BOLD: AEI3638, AEI3639, AEI3640, AEI3641), *T.formosana* (BOLD: AEN7193, AEN7194) and *Theopropus* sp. 1 WJ-2021 (BOLD: AEH8998, AEH8999) (Table 2).

A Taxon ID Tree was created in BOLD using the neighbor-joining (NJ) method following alignment based on K2P distances (Fig. 2). Each of the major clusters in the constructed K2P/NJ tree consisted of individuals from the same species, in general agreement with traditional taxonomy. The only exception was the specimen of *Hierodula* sp. BCM-2019, which was placed on a branch next to *H.chinensis*. The ASAP algorithm was the most conservative, lumping the sequences into 32 MOTUs, whereas a total of 58, 60, 68 MOTUs were delimited by jMOTU 17bp (~ 2.7%), sGMYC, and bPTP, respectively (Fig. 2). All species, except for *A.zhengi*, had their own unique MOTU or were assigned to more than one MOTU. The specimens of *A.zhengi* were split into two different MOTUs by all algorithms. Congeneric species were merged into the same MOTU in eight cases (Fig. 2). Additionally, one MOTU was shared by *H.chinensis*, *Hierodulalatipennis* Brunner, 1893, *Hierodulalonga* (Yang, 1997), *Hierodulazhangi* Wang & Dong, 1993, *Hierodula* sp. BCM-2019, *Rhomboderalatipronotum* Zhang, 1990, *T.formosana*, and *Titanodula* sp. WJ-2021, which represented a mixture of species from different genera.

The results of the jMOTU analysis using threshold values initially from 0 to 40 bp are shown in Fig. 1. A total of 57 MOTUs were determined by a 17 bp (~ 2.7%) distance cut-off. Four MOTUs were composed of different species of the same genus, viz., *Anaxarchagraminea* Stal, 1877 and *Anaxarchatianmushanensis* Zheng, 1985, *H.chinensis* and *Hierodula* sp. BCM-2019, *Statiliaagresta* Zheng, 1987 and *S.maculata*, and *T.aridifolia*, *T.sinensis* and *Tenodera* sp. WJ-2021 (Fig. 2).

The phenomenon of species being over-divided appears in the bPTP algorithm, with a total of 68 MOTUs divided (Fig. 2). *Acromantishesione*, *A.zhengi*, *C.gemmatus*, *Theopropus* sp. 1 WJ-2021, *S.agresta*, *Sinomiopteryx* sp. 1 WJ-2021 and *T.maculosa* were all divided into two MOTUs (Fig. 2, Table 2). Sixteen species were recovered as a single MOTU by all five species delimitation algorithms, viz., *Astyliasulamajor* (Beier, 1929), *Arriasticta* (Zhou & Shen, 1992), *Amantiswuzhishana* Yang, 1997, *Arria* sp. WJ-2021, *Caliris* sp. WJ-2021, *E.guttatipennis*, *E.yunnanensis*, *Gonypetabrunneri* Giglio-Tos, 1915, *Gonypeta* sp., *Leptomantellaxizangensis* Wang, 1993, *Leptomantella* sp. WJ-2021, *M.religiosa*, *Odontomantisplaniceps* Haan, 1842, *Odontomantis* sp. WJ-2021, *Pseudempusapinnapavonis* Brunner, 1893 and *Sinomiopteryx* sp. 2 WJ-2021 (Fig. 2). All algorithms except ASAP divided *Hierodula* sp. BCM-2019 and *H.chinensis* into a single MOTU, *S.occipitalis* was overclassified as four MOTUs, and *A.sinensis* was overclassified as three or four MOTUs.

Although the results of the sGMYC algorithm were similar to those of the jMOTU algorithm, it placed *A.graminea* and *A.tianmushanensis* into the same MOTU, *T.aridifolia* and *T.sinensis* were placed into the same MOTU (Fig. 2, Table 2). *T.aridifolia* and *T.sinensis* were pooled into an MOTU by bPTP, jMOTU and sGMYC analysis (Fig. 2). sGMYC and jMOTU were more highly concordant with morphospecies than other molecular species delimitation methods.

## ﻿Discussion

Most prior work on the mantis fauna of China only employed morphological approaches. Through large-scale COI-5P barcode sequencing, a reference library of verified barcodes has become an efficient tool for identifying unknown specimens in many insect groups. The utility of DNA barcoding has always depended on the taxonomic coverage of a reference library. The Chinese Mantodea fauna is remarkably rich with many endemic species. As of May 2024, only 61 barcode sequences representing five Chinese mantis species were in BOLD, so there is still a long way to go to attain full coverage. This study provided 318 COI-5P barcode sequences for 55 mantis species in China. These data helped improve the digital repository of barcode sequences, new COI-5P barcode sequences were generated, and 44 mantis species had not been previously processed. Two or more species are cryptic if they are morphologically similar, biologically different, and erroneously classified (and hidden) under the same species name (Bickford et al. 2007; Zhou et al. 2019). Morphological data alone often leads to overestimating or underestimating the actual number of species (Ahmadzadeh et al. 2013). DNA barcoding combined with species delimitation algorithms enables rapid distinction of morphologically similar species and detection of cryptic diversity within species (Zhao et al. 2022).

For most insect groups, barcode divergence lower than 2% often corresponds to intraspecific differences, while higher values reflect overlooked species recognized as distinct MOTUs (Huemer et al. 2020). The maximum intraspecific distances of five species (viz., *A.sinensis*, *A.zhengi*, *H.chinensis*, *S.occipitalis*, *T.formosana*) are higher than 3%, suggesting that further surveys are needed for these species to assess potential cryptic diversity. However, the divergence between young sister species may fall below the 2% threshold, while unusually variable species may exceed it (Ortiz et al. 2023).

The “BIN Discordance Report” analysis in BOLD is a tool used to validate the newly generated data, and members of a BIN usually belong to a single morphological species (Ratnasingham and Hebert 2013). BIN sharing might be explained by mitochondrial introgression following hybridization, recent divergence with or without incomplete lineage sorting, inadequate taxonomy, and misidentification (Kerr et al. 2007; Ward et al. 2009; Zangl et al. 2020; Geiger et al. 2021). The specimens of *H.chinensis* and *Hierodula* sp. BCM-2019 were merged in the same BIN (BOLD: AEI8831), and all other delimitation approaches agreed on the merge into an MOTU. Thus, specimens of *Hierodula* sp. BCM -2019 (unreliable identification) were considered as *H.chinensis*. Our Taxon ID Tree also shows this result. *Tenoderaaridifolia* and *T.sinensis* were merged into the same BIN (BOLD: AAW5350), both bPTP and sGMYC algorithms also classified them to a MOTU, and ASAP and jMOTU support their classification to a MOTU along with another two species from the *Tenodera* genus. Discordance between BIN assignments and morphological species may reflect the inability of barcode sequence variation to diagnose species because of introgression or their young age (Ortiz et al. 2023). DNA barcoding alone was insufficient to determine the validity of above mentioned *Tenodera* species.

One species was split into more than one BIN, and occurred as sister clusters on the barcode trees, often representing true potential cryptic diversity (Zhou et al. 2019). BIN assignments split *A.hesione*, *A.sinensis*, *A.zhengi*, *C.gemmatus*, *H.chinensis*, *S.occipitalis*, *T.formosana*, and *Theopropus* sp. 1 WJ-2021 into at least two BINs. For *S.occipitalis*, four BINs may reflect geographic clustering, in which BOLD: AEI3640, including specimens from Guangdong, BOLD: AEI3638 and AEI3641, including specimens from Yunnan, and BOLD: AEI3639, including specimens from Guizhou. For *A.sinensis*, four BINs also reflect geographic clustering, in which BOLD: AEI0969, including specimens from Guangdong, BOLD: AEI0967, including specimens from Guizhou, BOLD: AEI0968, including specimens from Hunan and Guangdong, and BOLD: AEN2989, including specimens from Guangxi. All delimitation approaches agreed on the subdivision of *A.zhengi* into two MOTUs. The BIN assignments of the above three species were matched to most other delimitation approaches, potentially implying detectable intraspecific diversity within *A.sinensis*, *A.zhengi* and *S.occipitalis*, or the probable existence of more than one species. The specimens of *T.formosana* were split into two BINs (BOLD: AEN7193, AEN7194) while they were recovered as a single MOTU by all other delimitation approaches. Based on the above results, a considerable number of cryptic species are likely awaiting description in Mantodea. The specimens of *S.agresta*, *Sinomiopteryx* sp. 1 WJ-2021, and *T.maculosa*, were recovered as two MOTUs each by bPTP analysis exclusively. Previous studies have shown that the delineation results of the bPTP method are inaccurate, and contain false positives (Luo et al. 2018; Song et al. 2018; Hofmann et al. 2019; Zhang et al. 2021). While under-sampling can exaggerate interspecific divergence, increased sample size and geographic coverage will decrease interspecific distances and expose species boundaries.

We employed both tree- and similarity-based approaches in delineating species to account for the limitations of each type of method (Hamilton et al. 2011; Esselstyn et al. 2012; Puillandre et al. 2012; Fujisawa and Barraclough 2013; Kapli et al. 2017). Different species delimitation algorithms were employed to associate morphological species with MOTUs, and in almost all cases, the nominal species were assigned as belonging to at least one MOTU. ASAP produced the most conservative results, with the highest number of merges, lowest number of splits, and the lowest OTU count. Both jMOTU and sGMYC algorithms produce more reliable partitions with the sampled morphospecies, while the bPTP and BIN algorithms typically resulted in an “overrated” solution. We used only the single-threshold version of GMYC because it has been shown to outperform the multi-threshold version (Fujisawa and Barraclough 2013; Talavera et al. 2013). Most of the MOTU splits detected in this study, such as *A.sinensis*, *A.zhengi*, *S.occipitalis*, were divided into at least two MOTUs, each using different species delimitation algorithms, except for the ASAP algorithm. Discordance between the boundaries of putative species is inferred by different delimitation methods, due to either over- or under-estimating the true number of lineages present in a morphologically defined species (Blair and Bryson 2017).

## ﻿Conclusions

This study provided 318 COI-5P barcode sequences for 55 mantis species in China. Of these, 44 species had not been sequenced before, thus being new COI-5P barcode sequences to science. Therefore, the current study represents an important step for the DNA barcoding of Mantodea in China. The specimens of *H.chinensis* and *Hierodula* sp. BCM-2019 were merged in the same BIN (BOLD: AEI8831) or MOTU. Thus, specimens of *Hierodula* sp. BCM -2019 were considered as *H.chinensis*. The MOTU splits may reflect cryptic/undescribed taxa, and if confirmed, the true species count for Chinese mantis could be higher than currently recognized. However, more detailed integrative studies combining nuclear markers and morphological characteristics are necessary for reliable identification.

## ﻿Additional information

### Author ORCIDs

Zhijun Zhou https://orcid.org/0000-0003-2625-6189

### Data availability

All of the data that support the findings of this study are available in the main text or Supplementary Information. All metadata are available in the BOLD project DNA barcode library for Mantodea of China (DS-DBMC).

## Supplementary materials

Supplementary material 1 Supplementary data Data type xlsx https://binary.pensoft.net/file/1260319This dataset is made available under the Open Database License (http://opendatacommons.org/licenses/odbl/1.0/). The Open Database License (ODbL) is a license agreement intended to allow users to freely share, modify, and use this Dataset while maintaining this same freedom for others, provided that the original source and author(s) are credited.Guangju Yang, Wenjing Wang, Yuwei Tong, Zhijun Zhou

Supplementary material 2 Supplementary image Data type tif https://binary.pensoft.net/file/1260320This dataset is made available under the Open Database License (http://opendatacommons.org/licenses/odbl/1.0/). The Open Database License (ODbL) is a license agreement intended to allow users to freely share, modify, and use this Dataset while maintaining this same freedom for others, provided that the original source and author(s) are credited.Guangju Yang, Wenjing Wang, Yuwei Tong, Zhijun Zhou
