# Supplementary figures and images for: ﻿Species delimitation and DNA barcoding for Chinese Mantodea (Insecta, Dictyoptera)
Source: Zookeys. 2025 Feb 24;1229:25–42. doi: 10.3897/zookeys.1229.129123 (PMC11876984; doi:10.3897/zookeys.1229.129123)

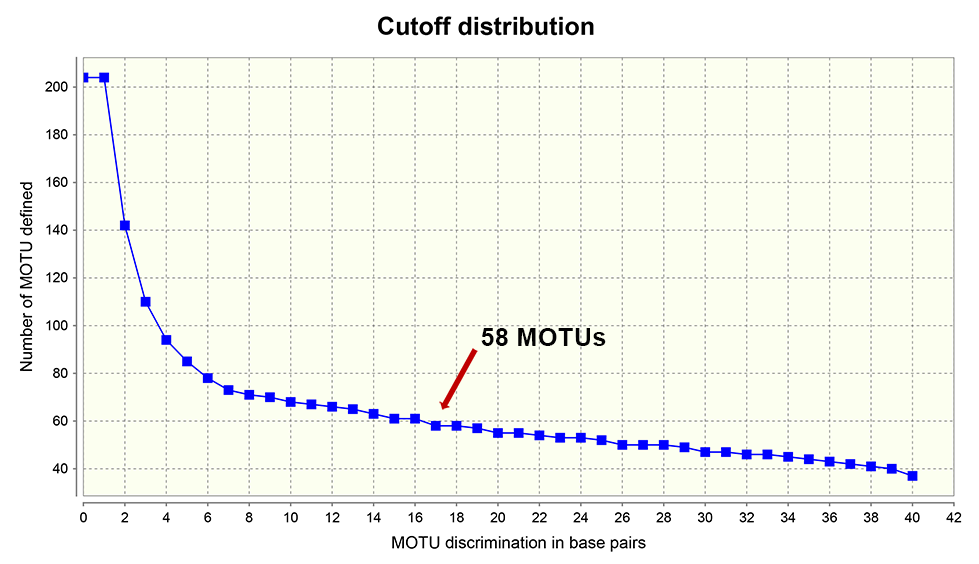

Supplement: Supplementary material 2 — Supplementary image [file zookeys-1229-025_article-129123__-s002.tif]
